# Diagnosis of Men-I Syndrome on ^68^Ga-DOTANOC PET-CT and Role of Peptide Receptor Radionuclide Therapy With ^177^Lu-DOTATATE

**DOI:** 10.5812/ijem.4313

**Published:** 2012-09-30

**Authors:** Santosh kumar Gupta, Suhas Singla, Nishikant A Damle, Krishankant Agarwal, Chandersekhar Bal

**Affiliations:** 1 Department of Nuclear Medicine, All India Institute of Medical Sciences, New Delhi, India

**Keywords:** Multiple Endocrine Neoplasia-I, Nuclear Imaging

## Abstract

**Abstract:**

MEN-I is a rare genetic disorder classically characterized by a predisposition to tumors of the parathyroid glands, anterior pituitary gland, and pancreatic islet cells. We present a case of MEN-I syndrome diagnosed using predominantly nuclear medicine imaging followed by radionuclide therapy, thus emphasizing the role of nuclear imaging in diagnosing and treating MEN-I.

## 1. Introduction

Multiple Endocrine Neoplasia-I (MEN-I) is an autosomal dominant disease with a broad spectrum of clinical manifestations (1, 2). Parathyroid hyperplasia, pancreatic neoplasia and pituitary tumors are the most frequent disease phenotypes, patients with MEN-I also infrequently develop subcutaneous lipomas, gastrointestinal, bronchial, and thymic carcinoid tumors (3, 4).

There is a very few case reports of the development of pancreatic neuroendocrine tumors (PNETs) in patients diagnosed with MEN-I syndrome related tumors. A carcinoid tumor in this case was located in the head of the pancreas, along with macroadenoma of the pituitary gland. This is the first paper to report the occurrence of PNETs in the association of a pituitary adenoma diagnosed by PET and treated with radionuclide targeting somatostatin receptors.

## 2. Case Report

**Figure 1:**
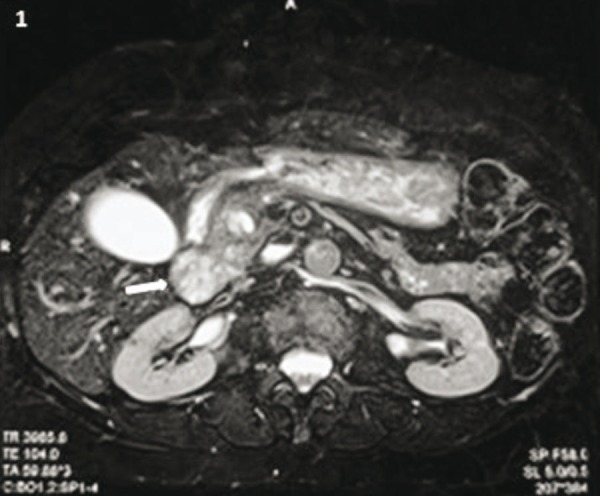
Trans-Axial T2-Weighted MR Image Shows Heterogeneously Hyperintense Lobulated Mass Lesion in the Head and Uncinate Process of the Pancreas (White Arrow).

71-year-old normotensive female, non diabetic presented with abdominal pain and increasing jaundice. Patient was admitted for evaluation and management. Endoscopic ultrasound revealed a periampullary mass measuring 5 x 5 cm in close proximity to the distal common bile duct (CBD). Fine needle aspiration cytology demonstrated metastatic deposits of a round cell tumor, morphologically resembling a neuroendocrine carcinoma. Magnetic Resonance Cholangiopancreatography performed for assessment of loco-regional disease revealed a lobulated mass lesion (2.7 x 2.3 cm) in the head and uncinate process of the pancreas (Figure 1).

As a palliative procedure, papillotomy was done and stent was introduced into the CBD during Endoscopic Retrograde Cholangiopancreatography. Hemogram, liver and kidney function returned to normal levels within a week after the procedure.

**Figure 2:**
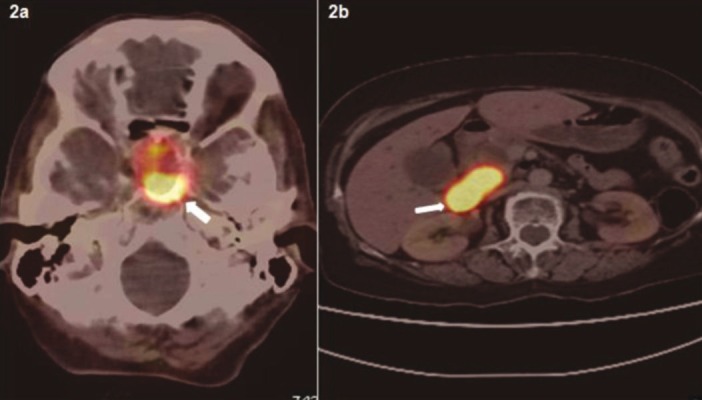
Trans-Axial Images of PET/CT With ^68^Ga-DOTANOC Demonstrate Abnormal Activity in Pituitary Macroadenoma and Pancreatic Mass.

**Figure 3:**
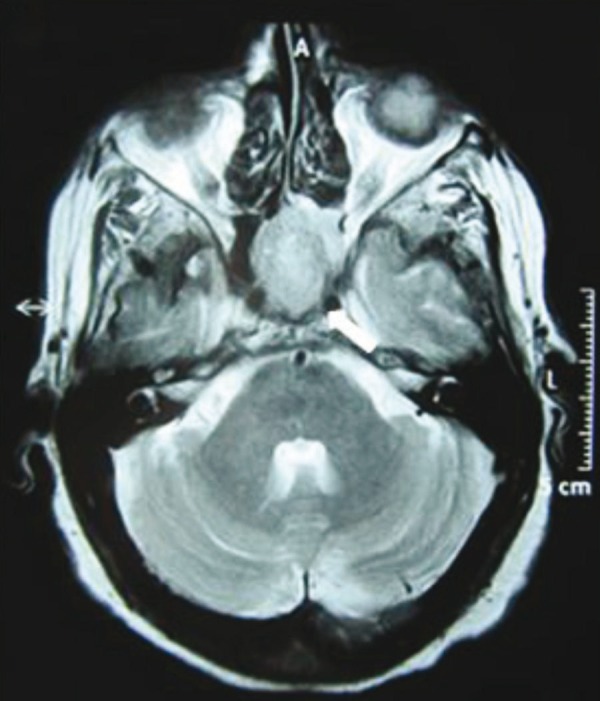
Trans-Axial T1-Weighted MR Showing Heterogenous Contrast Enhancement of Pituitary Macroadenoma.

**Figure 4:**
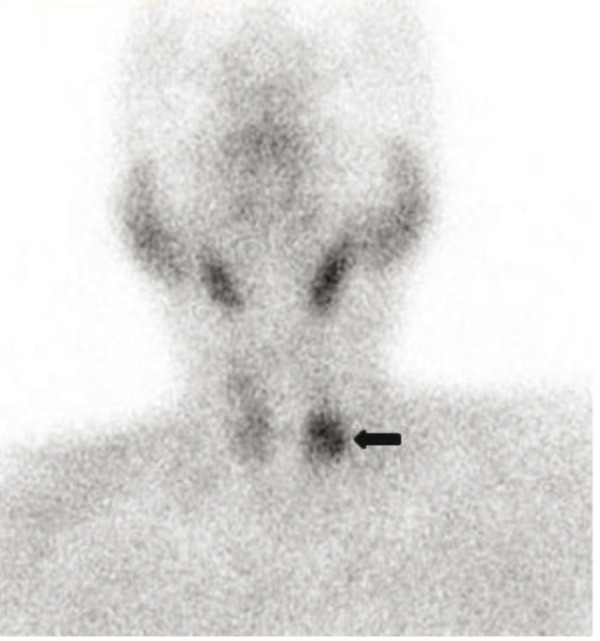
Planar Image of ^99m^Tc MIBI Scan Demonstrate Abnormal Activity in Parathyroid Adenoma (Black Arrow).

**Figure 5:**
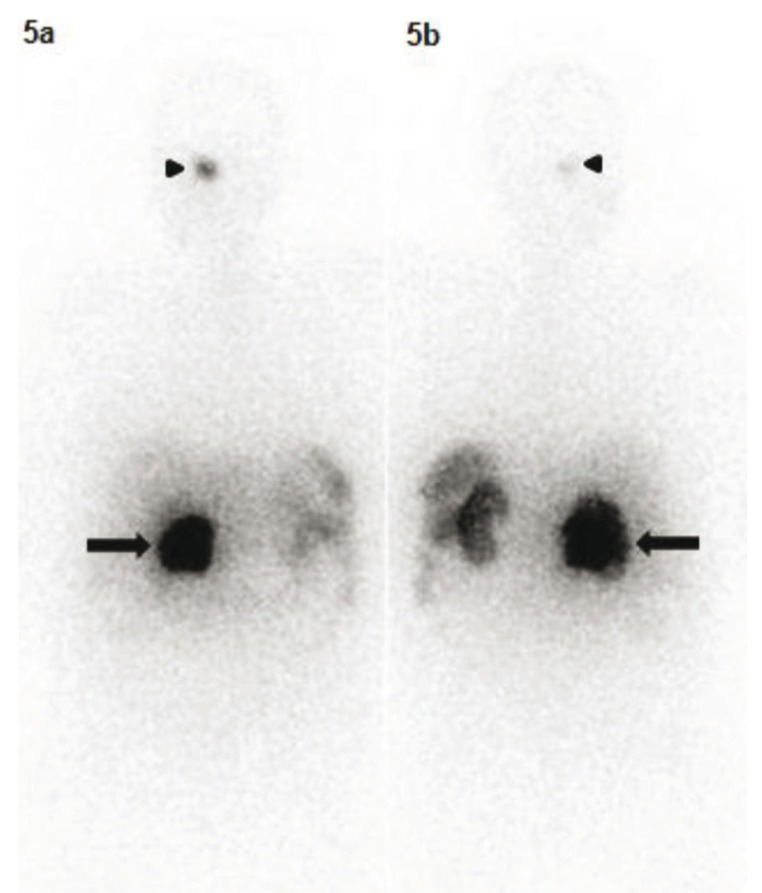
Anterior and Posterior Images of ^177^Lu-DOTATATE 24 hr Post- Injection Demonstrate Abnormal Activity in Pituitary (Black Arrow Head) and Pancreas (Black Arrow)

The patient atient was thereafter referred to the Nuclear Medicine Department for ^68^Ga-DOTANOC PET-CT study for staging and identification of distant metastases. There was evidence of somatostatin receptor expression on the tumor located in head of pancreas. Surprisingly, other intracranial lesion with intense radiotracer uptake was noted corresponding to enlarged pituitary gland (Figure 2a & Figure 2b). Serum Thyroid Stimulating hormone (TSH) and Prolactin were then ordered to investigate for functional status of the pituitary tumor, which were detected to be within normal limits. MR images confirmed the presence of pituitary macroadenoma (2.2 X 1.7 X 1.1 cm) which had herniated inferiorly through Sellar floor defect (Figure 3). Serum Parathyroid hormone (PTH) and ^99m^Tc-Sestamibi parathyroid scintigraphy was also ordered. The serum PTH levels were found to be elevated with focal radiotracer uptake in the region of the left inferior parathyroid gland (Figure 4). Biochemical markers and thyroid profile result are shown in Table 1. Serum TSH was found to be low with normal levels of T3 and T4 hormones. The patient opted for novel radionuclide treatment with ^177^Lu-DOTATATE which would simultaneously target pancreatic and pituitary lesions to avoid conventional surgical procedures. The patient was recruited for Peptide Receptor Radionuclide Therapy (PRRT) with ^177^Lu-DOTATATE and co-administered with the reno-protective amino acid mixture containing 25 g of Arginine and 25 g of Lysine. Post therapy whole body planar scans revealed intense uptake of radiotracer in head of pancreas and pituitary (Figure 5a and Figure 5b). Single administration of 150 mCi ^177^Lu-DOTATATE reduced serum Chromogranin A by 23%. The patient has been scheduled for next therapy 6 weeks later.

**Table 1:** Biochemical Markers Result

	Result b	Ref Range	Sensitivity	Precision	Assay Kit
Chromogranin A (pre-treatment)	660.34 ng/mL	< 100 ng/mL	10 ng/ mL	Intra- 8,9 %; Inter- 9,5 %	EIA a
Chromogranin A (post-treatment)	511.05 ng/mL	< 100 ng/mL	10 ng/ mL	Intra- 8,9 %; Inter- 9,5 %	EIA
PTH	104.6 pg/mL	15 – 68 pg/mL	≤ 5 pg/ mL	< 9%	CMIA a
T3 a (free)	3.09 pg/mL	2.3 – 4.2 pg/mL	0.05 pg/ mL	Intra - 4.9, 3.6%; Inter - 13.1, 7.9%	CLIA a
T4 a (free)	1.46 ng/dL	0.7 – 1.51 ng/dL	0.1 ng/dL	Intra - 2.17%; Inter - 4.28%	CLIA a
TSH a	0.459 µIU/mL	0.550 – 4.780 µIU/mL	0.02 µIU/ mL	Intra-15%; Inter- 20%	CLIA a
CA-19.9 a	42.05 U/mL	< 27.0 U/mL	0.2 U/ mL	Intra - 8.4,9.2; Inter -8.1,1.9%	EIA a
Prolactin	18.9 ng/mL	1.8 – 20.3 ng/mL	0.8 ng/ mL	Intra - 10%; Inter - 15%	EIA a

^a^Abbreviations: T3, 3,3, 5-triiodo-Lthyronine; T4, 3,5,3′,5′-tetraiiodo-l-thyronine; TSH, Thyroid-stimultating hormone; CA-19.9, Cancer Antigen 19-9); EIA, Enzyme Immunoassay ; CMIA, Chemiluminescent Microparticle Immunoassay; CLIA, Chemiluminescence Immunoassay

^b^All the hormonal or biochemical analytes are determined by using Enzyme-linked immunosorbent assay (ELISA)

## 3. Discussion

Neuroendocrine tumors (NETs) constitute a heterogeneous group of neoplasms with common characteristics and biological features. NETs were previously referred as APUDomas, by Pearse in 1968 when he described peptide secreting endocrine cells that share an ability to take up the precursors of biologically active amines, to produce active amine through subsequent intracellular decarboxylation, hence APUD (amine precursor uptake and decarboxylation) (5). Though the majority of NETs occur spontaneously, NETs may occur also as part of complex familial endocrine cancer syndromes such as MEN-I, MEN-2, Neurofibromatosis type 1 (NFT type 1), Von Hippel Lindau disease, and Carney's Complex (6-8).

MEN-I or Multiple Endocrine Neoplasia – Type 1, also called Wermer Syndrome, is a familial multiglandular endocrine tumor syndrome which can be variable in its presentation. The epidemiology of MEN-1 demonstrates a prevalence of 2-10 per 100,000 (9). It is 90% autosomal dominant inheritance, and is carried on the Menin gene on chromosome 11q13 (10). The clinical presentation of MEN-I is often remembered by the 3 P’s: parathyroid adenomas, PNET, and pituitary adenomas. In this case report, the patient demonstrated all three of these tumor types.

Pituitary tumors occur in approximately 40% of patients with MEN-I. These occur more commonly in women than men, and they are often more aggressive macro adenomas as compared to sporadic pituitary tumors. The most common type of pituitary adenoma in MEN-I is a prolactinoma (11). But nonfunctioning adenomas are also known. TSH levels tend to be generally low or normal in female patients with non-functioning pituitary adenomas, a finding consistent with our study. However, possible causes of subclinical hyperthyroidism should be ruled out whenever possible (12).

Pancreatic neuroendocrine tumors occur in the majority of patients with MEN-I (13). Nonsecretory neuroendocrine tumors tend to be large and are often very aggressive. Specific syndromes and clinical presentations are associated with the secretory types of neuroendocrine tumors: gastrinomas (severe gastric hyperacidity; Zollinger–Ellison syndrome), insulinomas (hyperinsulinism and fasting hypoglycemia), glucagonomas (diabetes and migratory necrolytic erythema), VIPomas (profuse watery diarrhea, hypokalemia, and achlorhydria; Verner–Morrison syndrome), Somatostatinomas (diabetes mellitus, steatorrhea, cholelithiasis).

Numerous radiotracers are available for endocrine tumor imaging, including ^99m^Tc-sestamibi, ^111^In-penterotide, ^18^-fluorodeoxyglucose (^18^F-FDG) and ^131^I-meta-iodobenzylguanidine (^131^I-MIBG). Each of these tracers has advantages and disadvantages. ^99m^Tc-sestamibi, for example, concentrates on cells with high mitochondrial content and is very useful in detecting parathyroid adenomas; it has a sensitivity of approximately 90%. ^111^In-penterotide is extremely beneficial in detecting NET because it acts on somatostatin receptors, making it highly tumor-specific. In contrast, FDG accumulates in any region with increased glucose metabolism, such as areas of inflammation, therefore making it less tumor-specific (14). Another drawback of using FDG for neuroendocrine imaging is that it classically causes false negative for carcinoid tumors (15).

Somatostatin Receptors (SSRs) are present in several organ systems and have an important function in the regulation of both endocrine and exocrine secretion. SSRs are expressed in the majority of tumors of neuroendocrine origin. So far five SSRs subtypes have been identified and cloned. Several somatostatin analogs have been successfully radiolabeled for diagnostic and therapeutic use. The relative usefulness of these radiolabeld somatostatin analogs is a function of their sensitivities and specificities of receptor binding and the characteristics of the associated radioisotope (16).

Positron emission tomography (PET) for imaging somatostatin receptor positive tumors is desirable because of its improved spatial resolution. ^68^Ga-labeled peptides show a rapid renal clearance and rapidly accumulate in the tumors (80% within 30 minutes). Concentration in tissues without expression of SSRs is low, providing higher contrast imaging. The activity administered in adults is 100-150 MBq and imaging is acquired 60 to 90 minutes after injection. ^68^Ga-DOTANOC that we have used being a PET tracer is an excellent tracer for neuroendocrine tumor imaging, and provides a high target-to-nontarget ratio that allows for the detection of very small lesions (17).

A new treatment modality for inoperable or metastasized PNETs is the use of radiolabeled somatostatin analogs. The majority of PNETs posses SSRs and can, therefore, be visualized using the radiolabeled somatostatin analog. A logical sequence to this tumor visualization in patients was to treat these patients with radiolabeled somatostatin analogs. Initial studies with high dosages of [^111^In-DTPA^0^] octreotide in patients with metastatized NET are encouraging, although partial remissions (PRs) were exceptional. This is not surprising because ^111^In-coupled peptides are not ideal for peptide receptor radionuclide radiotherapy (PRRT) because of the small particle range and, therefore, short tissue penetration of the Auger electrons (18). Kwekkeboom et al. compared the ^177^Lu-DOTATATE with [^111^In-DTPA^0^] Octreotide in patients with neuroendocrine tumors and found similar biologic half lives for both radionuclides, with providing a higher absorbed dose to most tumors by ^177^Lu (19).

One issue that we would like to emphasize is the benefit of ^68^Ga-DOTANOC PET-CT imaging in the workup of MEN-I. Information acquired from ^68^Ga-DOTANOC PET imaging may confirm or reveal the presence of a lesion, detect metastases from a primary tumor, or diagnose neuroendocrine conditions in which multiple lesions exist. In addition, because somatostatin-receptor expression is seen more frequently in well-differentiated tumors, visualization may suggest a more favorable prognosis. Patients with somatostatin receptors expressing tumors detected by ^68^Ga-DOTANOC PET are amenable to treatment with ^177^Lu-DOTATATE, particularly when conventional treatment is not feasible.

## 4. Conclusions

MEN-I is a rare familial multiglandular endocrine tumor syndrome. This case presentation describes the occurrence of all three major tumors of MEN1 (pituitary adenoma, parathyroid adenoma, and pancreatic neuroendocrine tumor) in a 71-year-old female.

Nuclear medicine diagnostic imaging namely ^68^Ga-DOTANOC scan is particularly helpful for identifying pituitary and pancreatic lesions in MEN-I. Uptake of ^177^Lu-DOTATATE on post therapy scan and decrease in serum chromogranin A after single administration indicate an alternative non-surgical option for treatment of patients with MEN-I.

## References

[1] Marx SJ, Vinik AI, Santen RJ, Floyd JC, Jr., Mills JL, Green J (1986). Multiple endocrine neoplasia type I: assessment of laboratory tests to screen for the gene in a large kindred.. Medicine (Baltimore)..

[2] Skogseid B, Rastad J, Oberg K (1994). Multiple endocrine neoplasia type 1. Clinical features and screening.. Endocrinol Metab Clin North Am..

[3] Burgess JR, Harle RA, Tucker P, Parameswaran V, Davies P, Greenaway TM (1996). Adrenal lesions in a large kindred with multiple endocrine neoplasia type 1.. Arch Surg..

[4] Shepherd JJ (1991). The natural history of multiple endocrine neoplasia type 1. Highly uncommon or highly unrecognized?. Arch Surg..

[5] Pearse AG (1968). Common cytochemical and ultrastructural characteristics of cells producing polypeptide hormones (the APUD series) and their relevance to thyroid and ultimobranchial C cells and calcitonin.. Proc R Soc Lond B Biol Sci..

[6] Duh QY, Hybarger CP, Geist R, Gamsu G, Goodman PC, Gooding GA (1987). Carcinoids associated with multiple endocrine neoplasia syndromes.. Am J Surg..

[7] Griffiths DF, Williams GT, Williams ED (1987). Duodenal carcinoid tumours, phaeochromocytoma and neurofibromatosis: islet cell tumour, phaeochromocytoma and the von Hippel-Lindau complex: two distinctive neuroendocrine syndromes.. Q J Med..

[8] Hough DR, Chan A, Davidson H (1983). Von Recklinghausen's disease associated with gastrointestinal carcinoid tumors.. Cancer..

[9] Yoshimoto K (2000). Multiple endocrine neoplasia type 1 : from bedside to benchside.. The journal of medical investigation : JMI..

[10] Lemmens I, Van de Ven WJ, Kas K, Zhang CX, Giraud S, Wautot V (1997). Identification of the multiple endocrine neoplasia type 1 (MEN1) gene. The European Consortium on MEN1.. Hum Mol Genet..

[11] Brandi ML, Gagel RF, Angeli A, Bilezikian JP, Beck-Peccoz P, Bordi C (2001). Guidelines for diagnosis and therapy of MEN type 1 and type 2.. J Clin Endocrinol Metab..

[12] Khalid S, Mahmood K, Arsalan M (2009). Serum Levels of FSH and TSH in nonfunctioning Pituitory Adenomas before and after Surgery.. Biomedica..

[13] Akerstrom G, Hessman O, Hellman P, Skogseid B (2005). Pancreatic tumours as part of the MEN-1 syndrome.. Best Pract Res Clin Gastroenterol..

[14] Groshar D, Bernstine H, Stern D, Sosna J, Eligalashvili M, Gurbuz EG (2010). PET/CT enterography in Crohn disease: correlation of disease activity on CT enterography with 18F-FDG uptake.. J Nucl Med..

[15] Groheux D, Hindie E, Tredaniel J, Giraudet AL, Vaylet F, Berenger N (2009). [PET-CT for evaluation of the solitary pulmonary nodule: an update].. Rev Mal Respir..

[16] Reubi JC, Schaer JC, Markwalder R, Waser B, Horisberger U, Laissue J (1997). Distribution of somatostatin receptors in normal and neoplastic human tissues: recent advances and potential relevance.. Yale J Biol Med..

[17] Maecke HR, Hofmann M, Haberkorn U (2005). (68)Ga-labeled peptides in tumor imaging.. J Nucl Med..

[18] Cremonesi M, Ferrari M, Bodei L, Tosi G, Paganelli G (2006). Dosimetry in Peptide Radionuclide Receptor Therapy: A Review.. J Nuc Med..

[19] Kwekkeboom DJ, Bakker WH, Kam BL, Teunissen JJ, Kooij PP, de Herder WW (2003). Treatment of patients with gastro-entero-pancreatic (GEP) tumours with the novel radiolabelled somatostatin analogue [177Lu-DOTA (0),Tyr3]octreotate.. Eur J Nucl Med Mol Imaging..

